# “Even We Are Confused”: A Thematic Analysis of Professionals' Perceptions of Processed Foods and Challenges for Communication

**DOI:** 10.3389/fnut.2022.826162

**Published:** 2022-02-23

**Authors:** Christina R. Sadler, Terri Grassby, Kathryn Hart, Monique M. Raats, Milka Sokolović, Lada Timotijevic

**Affiliations:** ^1^Department of Nutritional Sciences, School of Biosciences and Medicine, Faculty of Health and Medical Sciences, University of Surrey, Guildford, United Kingdom; ^2^Food, Consumer Behaviour and Health Research Centre, School of Psychology, Faculty of Health and Medical Sciences, University of Surrey, Guildford, United Kingdom; ^3^European Food Information Council, Brussels, Belgium

**Keywords:** processed food, food processing, classification system, thematic analysis, workshop, stakeholder views, risk communication, food system

## Abstract

Processed foods are increasingly under the spotlight since the development of classification systems based on proxies for food processing. Published critical reviews and commentaries suggest different views among professional disciplines about the definition and classification of processed food. There is a need to further understand perspectives of professionals on the conceptualisation of processed food and the agreements and disagreements among experts, to encourage interdisciplinary dialogue and aid communication to the public. The aim of this research was to elicit views and understandings of professionals on processed food, their perceptions of lay people's perceptions of the same, and their perspectives on the challenges of communicating about processed foods to the public. The online discussion groups brought together a range of professionals (*n* = 27), covering the fields of nutrition, food technology, policy making, industry, and civil society, mixed in 5 heterogenous groups. Through thematic analysis the following themes relating to the conceptualisation of processed food and challenges for communication were identified: (1) Broad concepts that need differentiation; (2) Disagreements on scope and degree of processing; (3) The role of food processing within the food system: the challenges in framing risks and benefits; and (4) The challenge of different perspectives and interests for risk communication. Throughout the discussions blurred lines in the characterisation of processing, processed foods, and unhealthy foods were observed. Participants agreed that consensus is important, but difficult. Participants identified a need for further interdisciplinary dialogue, including public engagement, to break down the observed issues, and work towards a mutual understanding and develop clear communication messages.

## Introduction

Food processing can be defined in different ways, such as referring to an action or procedure that alters the initial food, or the transformation of ingredients into food products (1, 2). Definitions vary in scope, either restricting food processing to methods “substantially altering the initial product” (1), or to those used by the food industry (3), or more broadly, to include simple techniques like washing and chopping (4). Processing food can serve different purposes such as preserving or enhancing nutritional content, making a food digestible, and/or safe to eat, prolonging shelf-life, and altering sensory aspects such as taste, texture, or colour.

Scientific interest in food processing has extended from the domain of food science and technology, within which it was formerly studied, to public health nutrition (3–5). Scientific papers and textbooks focus on understanding how combinations of processing methods and innovative techniques influence the physical, chemical, and (micro) biological properties of food. More recently, food processing has become a focus and a concern for public health scientists, with the emergence of the term “ultra-processed food” (3).

The development of dietary guidelines has evolved from a focus on nutrients to foods, food patterns, wider aspects of dietary behaviour, and resulting in “food-based dietary guidelines” (6). Some dietary guidelines, at first in Brazil (7) and now in other countries such as India (8), and regions such as Flanders in Belgium (9), refer to food processing and advise avoiding/limiting ultra-processed food. Hence the concept of “processed food,” and its classification, is increasingly in the spotlight stimulating debate among professionals within different scientific disciplines in the published literature (5, 10–13). In a recent critical review of classification systems, we found a lack of consensus on what features determine the level of food processing (14). We speculated how disagreements may stem from different perspectives and intentions, and we pointed to debates about the characterisation of food processing, nutritional assessment, dietary guidance and unhealthy foods.

While it is possible to glean such views through literature review, professionals' views on processed food have not yet been formally analysed, and a recent review highlighted this evidence gap, indicating a need for this interdisciplinary dialogue (15).

The aim of this research was to elicit views and understandings of professionals on processed food, their perceptions of lay people's perceptions of the same, and their perspectives on the challenges in communicating to the public about processed foods. We were interested in their views on where there are agreements and disagreements among experts, and on forming a consensus.

### Research Questions

- How do professionals conceptualise processed food?
- How do professionals perceive that the lay public conceptualise processed food?
- What are the main (dis)agreements perceived among professionals?
- Do professionals think consensus is important? If so, what is needed to achieve it?
- What do professionals think are the challenges in communicating to the public and how can they be overcome?

## Methods

### Ethics

The study was conducted according to the guidelines laid down in the Declaration of Helsinki and all procedures involving research study participants were approved by the University of Surrey Ethics Committee (FHMS 20-21 028 EGA). Written informed consent was obtained from all participants. Participants gave consent for video and audio recording of the discussion groups for the purposes of the research study, and for the use of anonymous verbatim quotations in publications. Comments made during the discussion groups are not attributable to individuals.

### Recruitment of Participants

Considering the multidisciplinary nature of the topic, we sought to invite a range of professionals to bring together a variety of perspectives. Representatives covering the fields of nutrition, food technology, policy making, industry, and civil society, were identified. Contacts were determined through group discussion and snowball techniques. Most of the identified professionals operated at an EU-level, but also included representatives at national and international level.

Based on limited published guidance on estimating sample size for focus group research (16), it was anticipated that five groups would provide sufficiently rich data. The study was not designed to provide an exhaustive overview of perspectives, or achieve consensus, but rather an informative and timely insight into professionals' views.

Lobe et al. (17) advises to limit the number of participants for online focus groups (ideally 3–5 participants per group). We over-recruited to offset potential non-attendance. A total *N* = 27 participants were recruited (nutrition = 6, food technology = 6, policy making = 5, industry = 5, civil society = 5; 40% male: 60% female).

Participants were attributed to a professional category (nutrition, food technology, policy making, industry, or civil society), for the purposes of mixing professionals in each group. However, the allocated professional categories were not mutually exclusive, for instance some participants identified themselves in another category reflecting dual-roles (e.g., nutritionist working in policy making). Participants were allocated to five *heterogenous* groups, based on stratified randomisation (according to the professional field assigned). Such heterogeneity within group discussions can “serve to uncover deeper insights” (18). Each group consisted of five to six participants. While it was not possible to truly balance gender due to the dominance of females recruited, each group had at least two male participants.

### Protocol

The synchronous focus groups were conducted online using the video communications platform *Zoom*, on 11 February 2021. In a plenary session, participants were first reminded how the data would be used, including confidentiality agreements, and encouraged to share their points of view. Participants were then put into their allocated breakout room, for 80 min. Each group was led by a facilitator, supported by a note-taker. The facilitators had received training and a briefing prior to the event.

The group discussions followed a structured format. Each group commenced with a round of self-introductions, where participants were also asked to share their motivation for joining. Each facilitator then led their respective group through the following discussion questions:

1a) What do you as professionals understand processed food to be?
1b) What do you think the lay public understand processed food to be?
2a) What and where do you think are the main agreements among professionals?
2b) What and where do you think are the main disagreements among professionals?
3a) Is consensus important?
3b) If so, what do we need to achieve this?
4a) What are the challenges in communicating to the public?
4b) How can we overcome them?

Discussions were further facilitated by use of an online collaborative tool, Padlet (Wallwisher, Inc.^1^), which is akin to a noticeboard using digital post-it notes. The questions appeared horizontally along the top of each Padlet board and participants were invited to add written responses in posts below each question. Participants could then express agreement or disagreement by rating the responses using the thumbs up/down buttons. For the purposes of encouraging discussion, for each question, the facilitator and note-taker helped the group to write a statement to reflect the main points of agreement. Following the discussions one participant sent additional inputs by email, expressing technical difficulties using the Padlet, in addition to general feedback points from other participants.

### Analysis

The data were analysed using reflexive thematic analysis informed by Braun and Clarke (19, 20), guided by a constructivist epistemology. Recordings from the five groups were transcribed verbatim, excluding non-verbal sounds. To anonymise the data, names were replaced with codes. The anonymised data were uploaded to NVivo 12 (QSR International Pty Ltd). To aid familiarisation with the data, the transcripts were printed, re-read and annotated with initial reflections. Padlets were consulted to aid interpretation of the discussion and cheque for alternative views. The principal researcher (CS) coded each transcript on Nvivo, using a combined inductive and deductive approach; coding was both data-driven and informed by prior research/knowledge, this included searching the dataset for re-occurring codes. Codes were then grouped into higher-level themes to reflect patterns of meaning in the data, while simultaneously checking the underlying data and self-reflecting on interpretations of the data. The collaborating researchers (MR, TG, LT, KH) reviewed 1-2 transcripts each and collectively reflected on the initial themes to enrich the reading of the data.^2^ The consistency of the themes was reviewed and revised in relation to the research questions and coding. The themes move beyond what people said (semantic content) and consider the assumptions underpinning the data (latent content). The themes were refined during the write-up, and relevant anonymised quotes were selected to illustrate the discussion.

## Results

**Figure 1:**
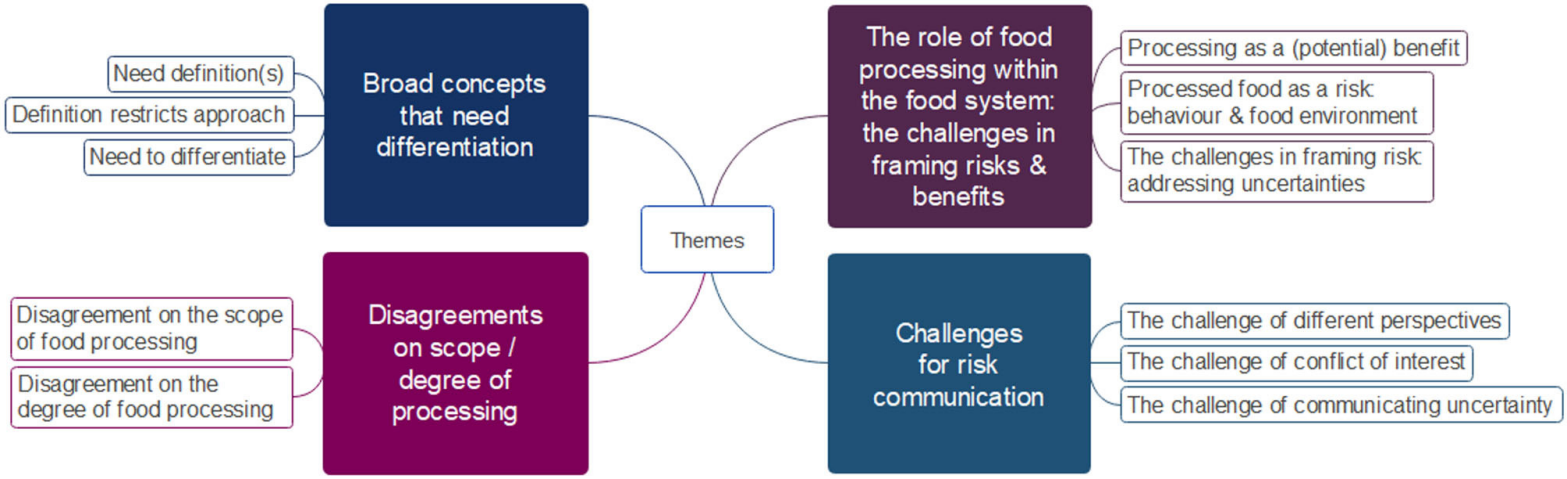
Themes generated from group discussions about the conceptualisation of processed food, agreements and disagreements among professionals, and the challenges in communicating to the public.

The topic of processed food prompted a wide range of discussions among participants. The dialogue oscillated between the impacts of food processing, identifying unhealthy foods and health promotion, which suggests tensions between the concepts of processing, processed foods, and health. The main themes developed based on the group discussions are presented below (Figure 1 provides an overview).

### Broad Concepts That Need Differentiation

*There appeared to be some agreement that processed food and food processing are broad concepts and need differentiation, although perhaps for different purposes. For instance, for differentiating processing methods/impacts or for distinguishing foods associated with negative health impacts*.

While participants gave varied descriptions of “processed food,” it was recognised that professionals tend to have a broad conceptualisation of the term, for instance, any change made to a food from its natural state, but there was acknowledgement that this breadth may be unhelpful in terms of research or communication.

*P27 “…from a more scientific viewpoint of course if we define it this way then all foods would be in the same category, more or less. So, as researchers we are forced to create a kind of differentiation between the different types of food processing. So we can't define processed foods as really foods to which processes have been done […] But I agree food processing for sure, is anything you do to a food. Yep.” (Group 5)*.

Considering the range of definitions, some participants insisted on a need for agreed (evidence-based) definitions. However, on the contrary, a “universal definition” was thought to be restrictive and unhelpful, suggesting that a rigid focus can distract from the important issues or goals.

*P4 “I think we have to be very careful with universal definitions, that's actually why I put definitions, because I think there's a need not to constrain either the thinking, the communication or the research around the issue, because we recognise that processed foods are a thing but we're not necessarily concerned about all processed foods. And therefore the concern or the messaging around those processed foods might depend upon the definition of the thing that you are concerned with. So I'm uncomfortable with one agreed universal definition because … the mischief in me will go and find an example that doesn't fit the definition.” (Group 1)*.

*P4 “It constrains the whole- It constrains the process and then you start worrying about the definition instead of the issue.” (Group 1)*.

Another participant argued that the function of the term “processed food” and the meaning assumed in everyday conversation differs to the dictionary definition. The participant explained, when they talk about “processed food” they do so in context of the associated health implications and the narrative around it; they do not wish to refer to all foods that have gone through processing.

Many participants acknowledged that a differentiation of the definition of processed foods may be useful, either for identifying ultra-processed foods, or in terms of defending processing (processing is not always “bad”). Some participants justified this based on the perception that lay consumers likely have a narrower view, and possibly think of more (ultra) processed, intensively manipulated foods, containing many e-numbers, or unhealthy foods. Thus, such communication would attempt to correct consumer misperceptions.

*P19 “Yeah I mean I was I was simply saying I think it is important to differentiate between processed, as in the simple processed food, cause most food has gone through some kind of a processing/process, and ultra-processed, because I think if we are talking about policy makers, if we're talking about consumers, there is a differentiation to be made and that's pretty clear from the 1b, everybody I think said more or less the same thing, that the lay public perceive processed food as being ultra-processed, unhealthy, manipulated in some way by industry, or actually manipulated intensively by industry not in some way, so I think it is. And if that's what we're going to be talking about that latter bit or are we talking about just normal process. I think we are going to be talking about ultra-processed, for want of a better phrase but, I think it is important to make that distinction, that's all. I would agree with what P9 said.” (Group 3)*.

*P9 “I cannot speak on behalf of the industry in general of course there are so many different aspects and positions, but I honestly think that it will actually help to have this distinction and to be able to explain what processed food is in general, so what we were saying. You do have to process food in order to consume it most of the times, and that does not necessarily have to immediately bring you to a McDonald's. So I think it's useful for the industry to have this difference, to be able to explain it better to consumers actually.” (Group 3)*.

However, a few participants disagreed on whether a classification or definition is even needed. A concern expressed by the participants was that defining a concept inevitably narrows down the possible framing, and thus restricts the decision-making approaches.

### Disagreements on Scope/Degree of Processing

*While many participants recognised a value in differentiating processing methods or processed foods, they perceived disagreements among professionals inherent in this classification, which potentially could relate to disagreements on what processing is and the impacts on health*.

#### Disagreement on the Scope of Food Processing

Discussions of what processed food is led to debates on the scope of food processing: what is and is not food processing and when does a process make a food a processed food?

There were mixed views on the boundaries of food processing. Some participants agreed that processing is “anything you do” to a food, for example harvesting, transporting or cleaning. One group argued that food processing is also done at home, rather than relating only to industrial processes. In contrast, one participant argued for separating the concept of “processed food” from the concept of “home cooking,” showing differences in perceptions of what defines the concepts.

Participants also questioned whether any processing makes a food “processed.” For instance, participants stated that it could be argued whether packaged fresh produce (e.g., an apple) would be deemed a “processed food,” because although packaging is a process, the food would be “unaltered.” One suggestion was to differentiate the term “food processing,” described as the transformative step, from the term “processed food.”

Several groups constructed “processed food” as multi-dimensional, contrasting processing to other dimensions notably nutrients and ingredients. The physical or integral properties of the original food was also recognised, although infrequently, as distinct from the nutritional properties. Participants indicated these different dimensions are a source of disagreement among professionals/scientists.

*P4 “I don't disagree with the second half of the statement, which is “many different forms of processing”, absolutely there are many different forms of processing. I disagreed with “the most” [most food is processed to some degree], but then I suppose it depends on what you define as processing. Because ultimately packaging you could argue is processing but in that you have not altered the food you simply put it in a package.” (Group 1)*.

*P21 “…traditionally we've been looking very much at individual nutrient aspects, but I think the research is moving a lot more too. It's not just about the nutrient composition of the products but it's also now the physical properties of the products. So, is the refined food coming from a food that still looks like a food or is it more coming from a chemistry department where the different elements are coming out of different bottles and put together, to resemble a food but actually there's no physical integral properties of any of the original foods. So, I think that's where there's disagreement between professionals.” (Group 2)*.

#### Disagreement on the Degree of Food Processing

The participants recognised the ongoing disagreement among professionals on the classification or ranking of different levels of processing or the “degree of processing,” and what is deemed acceptable or preferable in terms of processing. There was little explanation to understand how participants perceive what determines level or degree of processing. One participant advocated for the use of quantitative metrics. In some discussions, considerations moved beyond a technological assessment to also account for consumer needs–whereby concepts would serve a dual purpose for scientists and consumers.

*P5 “Yeah, generally I do agree, and I, from the scientists that I have spoken to at least, there seems to be an agreement that there needs to be a further distinguishing between the levels of processing. But then one of the main differences then occurs of how detailed this distinguishing in different categories should be. Should we go super detailed and then we enter into basically having a whole dictionary of different levels of processing, or should we be a bit more concise, to terms also make sense not only for scientific concepts but also to consumers later on?” (Group 5)*.

The relationship between “processing” and health, vs. other dimensions (e.g., ingredients, nutrients), is a source of disagreement. Some participants argued against the heuristic of equating degree of processing with degree of healthfulness.

*P17 “I think that it's really important for the public to find out about this complementary dimension of food related to processing, that is not the same one as the nutritional, as the nutrient profiles of the food. Because even in the scientific diaspora it's not something that everyone agrees on. So, it's even more and more complicated to the public to understand that if something is organic that doesn't make it healthy or, if something is less processed that doesn't make it healthy, or if something is highly processed that doesn't make it unhealthy. So, I think it's really important to communicate to people ways to disentangle these dimensions which is really complicated to do, and this is ?(a big)? challenge I think.” (Group 2)*.

While some participants appreciated that it may be useful to differentiate between processed and ultra-processed food, at the same time participants cautioned that consumers tend to view foods through the dichotomy of “good” or “bad.” The below extracted quote illustrates uncertainty as to whether ultra-processed foods are necessarily detrimental to health (cf. Section The Challenges in Framing Risk: Addressing Uncertainties).

*P25 “Yeah I just I think sort of looking forward and maybe I'm getting ahead of myself now is that it is, when you're thinking about communication to the public, it is important to make that distinction but also once you're then on ultra-processed food, is ultra-processed food always bad? Cause there's a lot of stuff you can do to food to make it better of course and there's a lot of work being done around that so that makes it really an interesting and very complicated discussion I think.” (Group 3)*.

The term itself was also described as contentious in some groups, particularly as the word, which contains a superlative (ultra), carries a negative connotation. Since the concept is reified to mean something inherently bad, it is viewed as potentially demonising of food processors even when it may be unwarranted. The term was also criticised for denoting something other than processing itself–the nutritional content.

### The Role of Food Processing Within the Food System: The Challenges in Framing Risks and Benefits

*The discourse pointed towards weighing-up the risks and benefits of processing for the food system and for consumers, in terms of food safety, food security, health and environmental impacts, and in the context of the determinants of food choice. Meanwhile some processed foods were perceived as a risk for vulnerable groups, particularly due to taste, accessibility, and marketing. Within these discussions there were clear challenges in framing risk and scientific uncertainty*.

#### Processing as a (Potential) Benefit

Throughout the discussions there was widespread agreement that food processing is important for food safety and food security i.e., providing safe foods and food supply. The role of processing in achieving sustainable food systems was also emphasised.

Considering the important role of processing, it was recommended to avoid communicating the message that processing is “bad.”

*P7 “…I think what we should agree on from a communication point of view, that you don't send any message like: processed is bad. […] -it is absolutely needed, so without any kind of processing there would be no life on earth, for sure […] in our society as we have it now. It's good to talk about processing, but actually we should much more discuss about what we want to aim…” (Group 2)*.

*P25 “…we've been talking about health a lot but as P15 just mentioned, sustainability, I think that also needs to come in, especially with a lot of the novel foods and trying to have meat replacements or bringing in insects or algae etc., it's very much about making those acceptable to the public, which is also processing, and then to make clear that this processing that we have done is not a bad thing.” (Group 3)*.

The above quotes illustrate the potential benefits of technological innovation in food processing for food sustainability and how these benefits may not be realised if processing is positioned as fundamentally detrimental. Benefits of processing were also illustrated through discussion of the trade-offs and potential consequences of not processing food: for instance, shorter shelf-life, food waste and higher food prices. One participant advocated for clear documentation of the trade-offs made in risk-based decision making.

To a lesser extent, the roles of processing in nutritional health and achieving healthier diets and meeting consumers needs were also noted. In defence of industrial processing, some participants contrasted this with home processing–for example the use of similar ingredients, the potential for increased preservation of nutrients, and the extent of regulation in terms of food safety. Improving taste and texture were briefly recognised as other important reasons for processing. There was some discussion about the practical challenges in preparing and eating fresh foods, and how a consumer's lifestyle demands may constrain their food choices. From this viewpoint, processing can sometimes make achieving a healthier diet more feasible.

At the same time, (unhealthy) processed foods, also referred to as “junk food,” were said to play a role in facilitating social inclusion, for example a child may feel stigmatised or excluded if he or she does not have a snack like the other school children. Hence, we identify that this perceived benefit (social inclusion) is in opposition with the health risks of over-consumption of such products which could lead to health inequalities.

The discussion points above align with viewing processed foods through the lens of the “theories of practice,” focusing on the benefits of food processing in the context of everyday practices such as working and shopping. Participants maintained that understanding the benefits of food processing must contend with the role it plays in facilitating and supporting these interlocking everyday practices.

*P11 “I think there's a need for more, a better mutual understanding and clarification of what we would like to achieve. And then for me it's all within, -the ultimate goal is to have a sustainable food system, so what does it mean, what do we need to achieve this. And that will be very different in various regions of the world, and for very different parts of the population, people who can afford to cook their meals from raw vegetables, other people don't have the time nor the money. So, there's so many needs, so then the question is how best to address the needs and then to identify an acceptable role of processing….” (Group 2)*.

*P27 “…but there are many other variables and I think time, convenience, y'know, if I'm gonna buy fresh stuff, I need to spend an hour or two cooking it. I've got to work, I've got all sorts of things to worry about, so it's much more complex than simply affordability, or accessibility in, in a country let's take like Belgium, and the problem may be quite different in different parts of this very country or in other countries. So, I think generalising there is, may lead us to not quite the right conclusions always.” (Group 5)*.

#### Processed Food as a Risk: Behaviour and Food Environment

Participants discussed food processing risks stemming from consumer's food behaviour and environment: relating to the influence of taste on eating behaviour, the limits of information-giving and the role of food system factors like food availability/access, cost and marketing.

Palatability of processed food was identified as a risk, suggesting that an enjoyable taste can override interest in health or willpower to eat healthy. We interpreted this to mean that the risks concern not only those pertaining to the food itself, but also to the way in which food processing interacts with consumer's eating behaviour.

Furthermore, we interpreted that the risks of processed food also relate to the way in which the food system is organised, which may amplify negative health effects and augment risks. Cheaper (and tastier) processed products such as crisps/chips were suggested as a determinant of malnourishment and obesity. There were discussions about disadvantaged populations and *access* to processed food; these groups were said to have a limited choice “between eating or not eating,” which was put forward as a challenge for communication. In turn, the accessibility of (ultra)processed foods may lead to health inequalities among vulnerable groups. Other participants argued that food choice is more complex than accessibility and affordability, highlighting other variables like time and convenience [as mentioned in Section Processing as a (Potential) Benefit].

While some participants advocated for food literacy, few pointed to the limited effect of information-giving on purchasing and eating behaviour. Furthermore, the marketing of (ultra)processed foods, and the relevant health and environmental impacts, were put forth as a potential source of disagreement among participants. A participant commented on the way in which business profits determine marketing, which competes with the promotion of fruits and vegetables. Another participant highlighted the huge advertising budgets of major food corporations and reach of communication, in contrast to public authorities.

These discussions suggest accessibility, affordability and marketing of less healthy processed foods are specific issues of concern.

*P4 “I think there are great trenches of our population that don't distrust processed foods, they don't give whether their food has been processed any thought because frankly they don't have access to anything other than processed foods, or consider the impact of those processed foods on their health because when you have the choice of eating or not eating, you choose to eat. I think there is certainly distrust amongst worried wealthy well, or those of us that are trying to eat healthily but I'm not sure that we're the problem.” (Group 1)*.

*P22 “…when we talk about public health and when we talk about nutrition, and y'know why are the impoverished populations, y'know, they're obese! They're malnourished but they're obese. Because it's so much cheaper, to just buy processed food–“processed” [AIRQUOTES] food and I say it in the sense of the word that the general population kind of understands it to be - altered in a way to make it more affordable, to make it tastier. It's so much cheaper to just buy a bag of y'know crisps, chips, than to buy fresh fruits and vegetables.” (Group 5)*.

#### The Challenges in Framing Risk: Addressing Uncertainties

There was a lack of agreement on how to frame the risks in terms of end-points, end-goals, and the combined impacts, as well as strength of evidence, and how to communicate scientific uncertainty.

Participants identified disagreements among professionals relating to the end-points for assessing risks of food processing and their relative importance. One group suggested a need for a better balance on “*metabolic safety*” vs. “microbiological safety.” The debates about the impacts of processing were identified as connected to a wider challenge—the unclear characterisation of “healthy” food. There was some advocation for discriminating between the, often confused, concepts of “healthy” and “safe.” Some participants also flagged the challenge of assessing and communicating about the healthfulness of individual products vs. the combination of foods in a diet.

*P17 “I completely agree with P21 on this, that the safety perception or the safety concept is totally different, depending on whether we are talking to a public health as- on a public health ?(aspect)? or we are talking to an association. Because I think that when we are, like, when we say ult- processed food or processing would make the food safe, an industrial or even a public health expert would say yes I do agree because thanks to processing we have microbiologically safe products, but then other people would argue ok but, safety is not just about microbiology, it is also about metabolism and how do we put the balance between microbiological safety and metabolic safety? And that's the main problem I think.” (Group 2)*.

Disagreements also related to the relative importance of different end-goals of food processing. One participant suggested that reasons like “food safety” and “food security” are sometimes used as an “excuse” for food processing, raising the question of whether the goal of processing needs to be delineated. This was contrasted to reasons like making a food more convenient, which could increase consumption (i.e., a risk). This perhaps carries the assumption that processing has a negative impact and needs to be justified, or that the purpose of processing, such as improving the palatability, may represent a risk in itself. Meanwhile, communicating the purpose of processing was recognised as a challenge, especially as the reasons for processing can be inter-connected.

Participants also alluded to the challenge of assessing the combined impacts of processing on multiple end-goals. For instance, the complexity of risk interactions in the food system and how these influence the concurrent goals of health and sustainability. One participant insisted the need for a life cycle assessment. There was an obvious dilemma regarding processed plant-based foods, with some participants suggesting these products may not support the goal of healthy diets. Yet, processed plant-based foods were presented as an example where both health and environmental impacts need to be weighed up in relation to dietary changes and societal goals.

*P6 “It's a bit like the current view of vegan food, when you actually look at a lot of the vegan and plant-based foods, the amount of processing that goes into those, is phenomenal. Far more than normal foods. So, is that still healthy?” (Group 4)*.

*P21 “…the issue is: is it better to have beef from hill grazing cattle and sheep or is it better to have refined plant-based foods. So there is an issue both from an environmental point of view and from a public health point of view.” (Group 2)*.

It was also identified that decision making includes broader considerations such as social economic impacts and cultural values, for example, which should be documented, along with the scientific uncertainties.

Participants identified that a potential root of disagreement may relate to the type and strength of evidence deemed sufficient for establishing facts: correlation or mechanistic models. Several participants advocated for an understanding of the mechanisms by which food affects health. For instance, they discussed whether it is the processing or the content of nitrates in processed meat that is responsible for causing cancer. One participant highlighted that the disagreement among scientists regarding the dimensions of nutrition vs. food processing may also relate to the strength of the evidence obtained. One participant highlighted that civil society or public health professionals are concerned with precautionary principles, i.e., taking action before uncertainty is resolved.

*P26 “…I think it's also difficult to communicate when you don't have any solid basis for what you say, so some element of y'know things that are established, I think there are still things that are established. Ok, maybe, then it's what do you consider established and for example, an arena is do you accept correlation as an establishment of the facts vs. a mechanistic approach ah that we do, I mean, generally in the food safety area now we try to look at mechanistic models. What makes such a molecule y'know toxic or not. Whereas, in the nutrition arena there is more, y'know, we look at broader studies that show a high degree of correlation but to me a correlation is not necessarily an explanation. But again, so, what do we consider, what is certain but there are still well established facts, and I think these are very useful to root a communication message.” (Group 4)*.

### Challenges for Risk Communication

*A dominant theme throughout the discussions various challenges for risk communication especially the recognition that different perspectives among professional groups is a challenge for clear communication. Conflict of interest, and uncertainty, were identified as additional complications when trying to reach consensus and communicate to the public*.

Considering the diverse perspectives and interests, a need for *constructive* dialogue was emphasised, directed towards a clear goal, to avoid getting caught-up in endless scientific debates on the details. To focus the discussions many participants agreed that consumer health should be the common end-goal, however sustainability was also mentioned. Participants identified a need for consensus on *what* messages need to be passed to the consumer.

#### The Challenge of Different Perspectives

The topic of processed food created confusion among participants. Confusion and disagreement among professionals were recognised as unhelpful for communication and consumer understanding, and amplified by opposing stories in the media. In this context, it was viewed that obtaining a consensus among professionals would be important for clear and consistent communication to consumers; benefits for research and policy makers were also mentioned.

A major theme in the discussions was that professionals have different perspectives, depending on their background and area of expertise or their professional motivations. Further interdisciplinary dialogue was recommended. However, it was repeatedly recognised that consensus would be difficult to achieve, and the participants expressed scepticism over its attainability. Some participants suggested that a more realistic aim may be a “mutual understanding” or a “consensus through disagreements.”

The topic of “processed food” was recognised as broad and complex, and one that can be discussed from many different angles, which need to be broken down. At times discussions veered off to broader topics relating to health behaviour, for instance drawing comparisons with smoking or promotion of fruit and vegetable intake. Un-packing the issues to identify the source of the disagreements and the evidence to move forward, which may in turn aid clearer communication, was recommended.

*P19 “I think my point about the clear messaging also echoes what P25 was saying, as well about what are we asking here, what is the point of the wider consultation, because it is a very wide topic and there are many stakeholders involved. There are many, many messages that you could give to the lay population, or to policy makers, or to fellow scientists, but if you give them all, they're all going to be lost. So that's where you need to, I think we need to be very clear about what it is we're trying to saying about ultra processed or processed foods. Are we just trying to improve the health literacy by improving the understanding of lay population of what we mean by processed foods or ultra processed foods? Are we trying to talk to them about the health benefits, or the lack of health benefits or some of them? Or are we trying to do something else? I think that's where I was coming from […] we need to understand what it is that we're trying to communicate […], and then that allows us to develop messaging around that, but I think it is such a wide complicated area […] it needs to be broken down I would say.” (Group 3)*.

*P15 “…I think that nutritionists and our health, public health experts, they don't know much about food processing, so, and they don't realise that almost every food that you consume is processed. So, and I don't think that food technologists, they understand food processing of course, they don't-, or probably don't have a full understanding of nutrition and health, but they understand food, I think.” (Group 3)*.

#### The Challenge of Conflict of Interest

Conflict of interest was identified as a challenge, since professionals have different concerns, which was recognised as inherent to a multistakeholder approach. For example, it is perceived that the intention of the food industry is to produce and market food products in order to sell more. Several participants commented that the food industry, as a result, is perceived as an unreliable source of information. However, condemnation of results or researchers linked to industry was also identified as a problem, since this cynicism easily dismisses work regardless of its merit. One participant said that while not all points-of-view are equally valid (e.g., opinions not supported by data), there should be equal opportunities to express those views and provide supporting evidence.

*P25 “…it's just that there are disagreements on, y'know, is there such a thing as ultra processed and what does it mean? And that may has to do with, y'know interests lurking in the background for various people […]”*

*P25 “I would have to ask if the others agree with that, because that's maybe a bit of a-”*

*P3 “I think this is, this is for everything, that's unavoidable. Sometimes it's intentional, sometimes it's not intentional, but it's like that.” (Group 3)*.

#### The Challenge of Communicating Uncertainty

There were some conflicting views on communicating scientific uncertainty. Where one participant cautiously advised against communicating without sufficient evidence, in contrast another advocated for continuous communication of research to consumers (and policy makers). Other participants recognised the importance of communicating honestly about (the evolving nature of science and) uncertainties–what we don't know and where science still evolves, for example the impact of food additives on the gut microbiome.

*P6 “…focusing just on the certainties, I mean there's a lot of evidence certainly in terms of sort of added sugars and particularly soft drinks etc. there's growing evidence on that, I think we have to be honest about that coming forward.” (Group 4)*.

How consumers handle risk and uncertainty was also identified as a challenge for communication. There was a perception that consumers regard foods as good or bad, yet food risks are not black or white. Some participants discussed the challenge of communicating the scientific nuances, and the risk of over-simplifying. In contrast, others discussed the fallacies of the knowledge deficit communication model: claiming that people do not necessarily need all the details to make the right decision. It was appreciated that consumers were not represented in the discussion groups, and public engagement would be a useful insight into lay understanding, wants and needs.

*P25 “…when you talk about involving stakeholders to have these discussions of course you also need to involve in some way, which is difficult, but you'd need to involve lay people.” (Group 3)*.

## Discussion

This study brought together a range of professionals interested in communication around processed food. The research aimed to elicit professionals' perspectives on the concept of processed food, identify and map the agreements and disagreements that exist about the issue of processed food among professionals, and the challenges for communicating to the public.

### Limitations

This study brought together a range of professionals in the fields of nutrition, food technology, industry, civil society and policy making, who had varying degrees of technical knowledge. To further pinpoint the agreements and disagreements relating to the degree of processing, for instance, it would be useful to gather inputs from technical experts. Furthermore, consumers were a missing stakeholder, consumer research and engagement would help understand the communication wants and needs of the public. However, it may be helpful to maintain a distinction between scientific needs and consumer needs.

Eleven participants indicated they had watched EUFIC's symposium on processed foods, which may have informed their views. In some cases, the participants were familiar with the facilitator and possibly each other, and this may have influenced the conversation. Thematic analysis is an inherently subjective process, involving analysis and interpretation by the researcher, hence we engaged in continual reflection on the assumptions and how these may influence the coding.

During the discussion groups we utilised an online noticeboard tool to assist interaction. While each group wrote statements to reflect the consensus for each question, we noted that the written statements did not always accurately reflect the conversation recorded. For example, statements were challenged, and the consensus was revised but not edited, perhaps due to a lack of time.

A clear limitation was the duration available for discussion, given the breadth of the topic and the complexity of the debate. The participants often used terms, such as “highly processed” without enough probing to clarify the underlying meaning and draw conclusions. The issues should be further broken down and discussed with a specific goal in mind, to limit the scope of the discussions. More directive questioning and probing could be used to support more concrete conclusions. Participants could also be asked to provide scientific evidence to underpin their arguments. An alternative approach could be to use the Delphi technique to construct consensus, which allows time for thoughtful consideration. Or, a multidisciplinary scientific committee could be convened, to jointly collaborate on evidence-based position statements.

### Area of Contention–Processing and How It Relates to “Healthy Food”

There are tensions between the concept of “food processing,” which is recognised as playing a role in food security and sustainability, and “processed food” which is synonymous with less healthy/natural foods.

Unsurprisingly, participants pointed to disagreements relating to the *degree of processing*, which is in line with our previous research comparing the basis of classification systems (14). The discussions gave greater context to these issues and provided a useful framing.

Notably, processed foods are viewed as multi-dimensional. There are disagreements relating to the distinction and relevance of ingredients and nutrients vs. processing, in the characterisation of “healthy food.” Furthermore, the boundaries of food processing are unclear. This follows the argument of Botelho et al. (5) to make a distinction between process and formulation (recipe). The term ultra-processed has previously been contested in the literature (13, 15, 21, 22), but may be/become a shorthand for explaining the complexity behind processing and food risk. The prefix ultra means “beyond what is ordinary, proper or moderate; excessively or extremely” (23). This would suggest that there is a norm for what is deemed a reasonable amount of processing, which participants indicated is not yet agreed upon. Furthermore, the ambiguity of the processing dimension, and the conflation with nutrients, and other elements, may solidify the misrepresentation rather than create clarity in risk communication.

Further work is needed to untangle and agree on the dimensions relating to the properties of food products and their relationship to health, and the implications for dietary guidance. The findings agree with a commentary provided by Julia et al. which highlights the lack of consensus on the dimensions and the challenges in defining the “healthiness” of foods (24). The role of dietary guidelines, which outline a healthy diet, is contrasted to that of food labelling, which indicates the relative healthiness of a food product; both approaches are deemed complementary (24).

Julia et al. consider that while nutrient content is currently the best available evidence to assess the impact of a food on health, emerging research may allow for other aspects to be accounted for in future. The authors recommend clear communication that nutrient-based labelling is a tool which provides relative information on nutritional content only, and they advise against dichotomising foods into healthy or unhealthy (24). At the same time, it is known that consumers tend to use heuristics, i.e., rules of thumb, based on simple cues, such as food packaging, to help make purchase decisions (24, 25).

Drewnoski et al. (26) found strong relations between the NOVA categorisation of processed foods and a nutrient density profiling system (Nutrient Rich Food Index). However, proponents of NOVA argue against nutritionism–the reductive focus on nutrients, and determination of the healthfulness of a product by nutrient content alone (22, 27). Labelling requirements have been seen to influence industry practices, for example by encouraging reformulation of products (28). However, there is potential for unintended consequences when nutrition labelling and reformulation efforts focus on selective nutrients-to-limit, and not the quality of ingredients (27). There could be further discussion on the limitations of algorithms used in labelling, and relations with food processing. Drewnoski et al. (29) recommend the modification of nutrient profiling models to incorporate food groups and dietary ingredients such as whole grains, to better align labelling with food-based dietary recommendations. This would make a distinction between refined carbohydrates, where the grain has been processed to remove parts, and whole grains which contain more nutrients and bioactives. The Nutri-Score algorithm, for instance, already awards points for fruits, vegetables, nuts, and olive/rapeseed oil (30). This is a step towards expanding the conventional nutrient-based approach to include food groups, which partially supports the intentions of NOVA.

A randomised controlled trial found that people consumed more energy and gained weight when provided (*ad libitum*) an ultra-processed diet, vs. an “unprocessed” diet (31). The mechanisms, which food properties cause excessive energy intake and negative health outcomes, are still under investigation (32). For example, it is plausible that sensory properties that make foods quick to consume, and a high energy density, increases consumption (33). Processing such as grinding and chopping can increase the metabolizable energy, i.e., the amount of calories absorbed from food, as shown with almonds (34). The highly degraded physical structure of ultra-processed foods, or other aspects such as additives, have been suggested as possibly relevant for the associations of ultra-processed foods with other health outcomes such as cardiovascular disease (35).

The inclusion of cosmetic additives are indicated as a key component of ultra-processed foods, not part of nutritional assessments, however existing safety assessments of additives and emerging research needs further debate, as previously discussed (14).

### Area of Contention–Food Security and Availability of “Healthy Food”

Food processing is represented as a double-edge sword for food security. Food processing can support food security by stabilising seasonal foods and thus making them available all year, but the availability (and low-cost) of (processed) food products associated with poor dietary patterns may put vulnerable populations at risk of health inequalities. The discussions among the participants suggest that food processing can both increase and decrease food risks, and suggest that many processed food products are less healthy or encourage unhealthy dietary patterns.

As explained in the above section, the characterisation of “healthy food” is contested. Studies show intakes of ultra-processed foods vary between countries, with the highest intakes in the US and the UK, where they contribute over 50% of energy intake (36).

Ultra-processed foods have been found to have lower nutrient density, higher energy density, and lower per calorie cost (37). Meanwhile, a study of food prices in Belgium found that individuals would pay a higher financial cost when following dietary recommendations, than not (38). For example, adult diets meeting recommended intakes of vegetables were 20% more expensive. Furthermore, the EAT-Lancet diet, which based on review of the scientific evidence aimed to provide a reference for what constitutes a healthy diet from a sustainable food system, has been deemed unaffordable for low-income populations (39).

A great number of food products sold in the EU were found to not meet criteria of nutrient profiling models intended to restrict food marketing to children (40). For instance, breakfast cereals tend to be high in sugars and low in fibre. Furthermore, products not meeting such criteria often have marketing directed to children, and health claims on the food packaging (41, 42). Price promotions also tend to be for less healthy food products (43). Furthermore, without the application of nutrient profiling, less healthy products can carry potentially misleading nutrient or health claims (44). This suggests that monitoring and incentives to reformulate products are needed to improve the nutritional quality of pre-packaged foods, in addition to food marketing policies.

### Area of Contention–Processing of Plant-Based Foods

Plant-based food products are debated both in terms of the healthiness of the product, and tensions between health and sustainability goals. Processing of these foods is viewed both positively and negatively.

These products can be contrasted to the original (whole) plant foods (e.g., veggie-burger vs. pulses and legumes), and to animal protein sources (e.g., veggie-burger vs. beef burger). Plant-based food products can contain high levels of added salt, saturated fat and/or sugar, and are often formulated with extracted ingredients, such as plant protein isolates (45). Santo et al. state that it is currently unknown whether substitutes derived from plant protein isolates offer nutritional or disease-risk reduction benefits comparable to whole legumes (46). The impact of these products also depends on which foods they replace and the associated dietary patterns (46). Tso and Forde modelled switching animal- to plant-based products, highlighting this could potentially reduce the nutritional quality of diets (45). For instance, products such as burgers may be eaten with a refined bun and fries, with minimal vegetables (46). This may also be of concern because the known health and sustainability benefits of plant-based diets may provide a “health halo” for processed plant-based products despite an unfavourable nutrient profile, and unknowns regarding whether processed ingredients offer the same health benefits as (whole) plant foods (45, 46). Such products may also contain food additives, some of which may not be tolerable to some consumers, for example by potentially causing adverse gastrointestinal reactions (46).

### Possible Reasons for Contention

The results show how the topic of processed food can be viewed from multiple angles and highlight the ambiguity and confusion surrounding the concepts of food processing, (ultra)processed food, and healthy food. Often the conversations jumped between concepts, for instance discussing food processing when defining processed food, or discussing health behaviour when talking about communicating the impacts of processing, suggesting blurred lines between the concepts.

The boundaries of the “processing” and “nutrition” dimensions of food can overlap, depending on the perspective. For example, when added ingredients such as fat, sugar and salt, influence the degree of processing this overlaps with existing energy and nutrient-based product evaluations. However, nutrient profiling models used for labelling do not fully capture nutrient density, which is indeed much wider than fat, sugar and salt (47).

The negative connotations of processed food may trigger food scientists to defend the positive role of processing for food safety and security. Meanwhile public health professionals are also concerned about broader aspects than the processing methods, ingredients, or nutrients–including marketing and eating behaviour. We suggest that it could be helpful to disentangle the risks of “processed foods” relating to the food environment (e.g., cost, availability, marketing) from properties integral to the food.

Classifications intended for both research and communication–for investigating the health effects of processes and for advising consumers on healthier products–may also be a source of confusion and exacerbate tension between scientific nuance and over-simplification.

It is interesting that the perceived mismatch between public and expert representations of the term “processed food” triggered some support for differentiating it from “ultra-processed food.” Such an approach is rooted in social representations of the issue. Literature now recognises that experts, like the lay public, perceive risks subjectively, based on their social experiences and worldviews, in addition to objective scientific appraisals (48). Participants may have been influenced by the framing of the discussion questions which may have prompted comparisons of professional and lay understandings. Among participants advocating for a taxonomy there was little discussion about the technical parameters that separate the two food groups, perhaps because participants lacked technical knowledge.

### Implications of Findings: Potential Areas for Consensus Seeking

It was paradoxical that participants identify a need for consumers to understand the nuances about food processing and healthy diets, yet disagree and are uncertain as to the messages to pass on. Conflict and disagreement among professionals could sow doubts and amplify consumer confusion about this topic, leading to either (a) amplified or attenuated perception of risk; (b) loss of trust; (c) rejection of any messages (49). Public scepticism in science is particularly evident in relation to certain topics, recently made salient with societal discussion on COVID-19 (50). Contradictions and debates between experts, and conflicting media coverage, can create confusion about dietary recommendations and can erode public trust and confidence in science (51). In contrast, communicating expert consensus can positively influence attitudes and support for policies on societal issues, according to the *Gateway Belief Model* (52).

Guastafson and Rice distinguish different types of uncertainties: knowledge gaps (deficient uncertainty), shortcomings of the research (scientific uncertainty), error or probability ranges (technical uncertainty), and controversy among scientists (consensus uncertainty) (53). While communicating *consensus uncertainty* is likely to have negative effects, conveyance of deficient, technical, or scientific uncertainties can result in positive or neutral responses (53).

Hence it is important to communicate the scientific certainties, and to explain to the public the ongoing scientific (or deficient or technical) uncertainties, and the processes of decision making among the professionals. Obtaining consensus might be difficult, but it is important for scientific progress and public trust.

A first step could be to identify and agree the scientific certainties in relation to the debates regarding food processing and processed food. There are a growing number of studies providing associations between foods broadly categorised as “ultra-processed food” and negative health outcomes. Despite disagreement on the terminology, many of these foods are energy dense, and high in saturated fat, sugar and salt (26), which based on participant's views, could be agreed upon. Meanwhile there is strong evidence to limit specific processed products such as sugar-sweetened beverages (54). However, we predict there could be ongoing disagreement on whether it is nutrients-to-limit that are the problem, or *processing*–reducing/removing beneficial nutrients-leading to less nutrient-dense foods, in addition to the role of non-nutrient food properties. This also leads to questions of how to communicate about the role of such foods in a diet.

As explained above, studies researching the food properties beyond nutrients which could be responsible for the negative health associations of ultra-processed foods are needed (32).

There is consensus that food processing has a role to play in the food system. The discussions aligned with a food systems approach, a holistic analysis of how the interactions between food production, processing, distribution and consumption, contribute to the concurrent health and sustainability goals (55). For instance, food processing can be used to reduce food waste, for example by valorising processing by-products and allowing safe food storage and transportability (13, 56). Furthermore, exploring this issue from the lens of practices goes beyond individual food choice and explores the systemic determinants and factors influencing people's engagement with and sense-making about food processing and food products. The meanings, materials and competences of practices such as shopping, cooking and eating are intertwined with the constraints of other practices such as parenting or caring responsibilities, working and transportation (57). Looking at social practices could help reframe problems regarding processed foods by honing-in on the specific issues, for instance, cost, availability, food preparation skills, access to equipment, time available. This approach has been explored for issues related to processed food–such as cooking (58), snacking (59), convenience food (60), inequalities in healthy eating (61) and promotion of sustainable food consumption (62). Another question is also how to find a balance, or manage the trade-offs, between food security, nutrition, and sustainability. The UK's Academy of Nutrition Sciences has called for the combining of full nutritional composition with environmental metrics in life-cycle assessments of foods (63).

Further interdisciplinary dialogue is required to disentangle the dimensions relating to properties of “processed food” and seek consensus on the relevance and classification of “degree of processing.” In addition, there is a need to align on the scientific certainties and uncertainties and the communication messages for the public. While striving towards a mutual understanding, experts should be mindful to not let nuanced debates on terminology distract from important public health and environmental goals. Transparency regarding potential conflicts of interest, and appropriate governance, will be important. Identifying the root issues and viewing food processing as part of a complex food system, is needed to understand how processing can be optimised towards the goal of equitable, safe, sustainable, and healthy diets.

## Data Availability Statement

The datasets presented in this article are not readily available because while the data have been anonymised, they still contain information which could potentially identify individuals which would need to be removed. Requests to access the datasets should be directed to Christina Sadler, c.sadler@surrey.ac.uk.

## Ethics Statement

The studies involving human participants were reviewed and approved by University of Surrey Ethics Committee. The participants provided their written informed consent to participate in this study.

## Author Contributions

CS: conceptualisation, methodology, investigation, writing–original draft, writing-review and editing, and visualisation. TG, KH, MR, and LT: conceptualisation, writing-review and editing, and supervision. MS: conceptualization and supervision. All authors contributed to the article and approved the submitted version.

## Funding

This work was supported by the University of Surrey Doctoral College and the Faculty of Health and Medical Sciences [Studentship Award/Faculty-Funded Award]; and the European Food Information Council.

## Conflict of Interest

CS (part-time) and MS (until March 2021) were employed by EUFIC, which in 2020 received a third of its funding from the food and drink industry. TG supervises PhD students partially funded by Mondelez and McCain Foods Ltd. MR and LT's research centre has provided consultancy to and received travel funds to present research results from organisations supported by food and drinks companies. The funders had no role in the design, execution, interpretation, or writing of this study. The remaining author declares that the research was conducted in the absence of any commercial or financial relationships that could be construed as a potential conflict of interest.

## Publisher's Note

All claims expressed in this article are solely those of the authors and do not necessarily represent those of their affiliated organizations, or those of the publisher, the editors and the reviewers. Any product that may be evaluated in this article, or claim that may be made by its manufacturer, is not guaranteed or endorsed by the publisher.

## Footnotes

^1^ https://padlet.com/

^2^This research benefited from a research team with diverse professional expertise. The lead author was a postgraduate researcher with a background in nutritional sciences and experience in science communication. The second author was a lecturer in food science with a background in chemistry, and interests in plant biology and nutrition. The third author was a registered dietitian and senior lecturer, with interests in nutrition and dietary assessment. The fourth author was Professor and Director of the Food, Consumer Behaviour and Health Research Centre with expertise is in the area of public health and behavioural nutrition research. The fifth author was a principal research fellow, Deputy Director of the Food, Consumer Behaviour and Health Research Centre, and associate fellow and chartered psychologist, with interests in stakeholder engagement and dialogue in policy and science and risk perception and governance.
